# Transient CD4^+^ T cell depletion during suppressive ART reduces the HIV reservoir in humanized mice

**DOI:** 10.1371/journal.ppat.1011824

**Published:** 2023-12-06

**Authors:** Lijun Ling, Chandrav De, Rae Ann Spagnuolo, Nurjahan Begum, Shane D. Falcinelli, Nancie M. Archin, Martina Kovarova, Guido Silvestri, Angela Wahl, David M. Margolis, J. Victor Garcia

**Affiliations:** 1 International Center for the Advancement of Translational Science, University of North Carolina at Chapel Hill, Chapel Hill, North Carolina, United States of America; 2 Division of Infectious Diseases, Department of Medicine, University of North Carolina at Chapel Hill, Chapel Hill, North Carolina, United States of America; 3 Center for AIDS Research, University of North Carolina at Chapel Hill, Chapel Hill, North Carolina, United States of America; 4 UNC HIV Cure Center, University of North Carolina at Chapel Hill, Chapel Hill, North Carolina, United States of America; 5 Department of Microbiology and Immunology, School of Medicine, University of North Carolina at Chapel Hill, Chapel Hill, North Carolina, United States of America; 6 Yerkes National Primate Research Center, Emory University, Atlanta, Georgia, United States of America; 7 Department of Pathology and Laboratory Medicine, Emory University School of Medicine, Atlanta, Georgia, United States of America; 8 Department of Epidemiology, Gillings School of Global Public Health, University of North Carolina at Chapel Hill, Chapel Hill, North Carolina, United States of America; Vaccine Research Center, UNITED STATES

## Abstract

Lifelong treatment is required for people living with HIV as current antiretroviral therapy (ART) does not eradicate HIV infection. Latently infected cells are essentially indistinguishable from uninfected cells and cannot be depleted by currently available approaches. This study evaluated antibody mediated transient CD4^+^ T cell depletion as a strategy to reduce the latent HIV reservoir. Anti-CD4 antibodies effectively depleted CD4^+^ T cells in the peripheral blood and tissues of humanized mice. We then demonstrate that antibody-mediated CD4^+^ T cell depletion of HIV infected ART-suppressed animals results in substantial reductions in cell-associated viral RNA and DNA levels in peripheral blood cells over the course of anti-CD4 antibody treatment. Recovery of CD4^+^ T cells was observed in all tissues analyzed except for the lung 26 days after cessation of antibody treatment. After CD4^+^ T cell recovery, significantly lower levels of cell-associated viral RNA and DNA were detected in the tissues of anti-CD4 antibody-treated animals. Further, an 8.5-fold reduction in the levels of intact HIV proviral DNA and a 3.1-fold reduction in the number of latently infected cells were observed in anti-CD4-antibody-treated animals compared with controls. However, there was no delay in viral rebound when ART was discontinued in anti-CD4 antibody-treated animals following CD4^+^ T cell recovery compared with controls. Our results suggest that transient CD4^+^ T cell depletion, a long-standing clinical intervention that might have an acceptable safety profile, during suppressive ART can reduce the size of the HIV reservoir in humanized mice.

## Author summary

Current long-term antiretroviral therapy does not eradicate cells latently infected with HIV. Latently HIV-infected cells are essentially indistinguishable from uninfected cells and cannot be easily targeted by currently available strategies. CD4^+^ T cells represent the most well-characterized and largest HIV reservoir. Anti-CD4 antibody mediated CD4^+^ T cells depletion has been proved to be reversible and well-tolerated in humans. In this study, we evaluated the effect of transient depletion of human CD4^+^ T cells with an anti-CD4 antibody on the size of the HIV reservoir in HIV-infected ART-suppressed humanized mice. CD4^+^ T cells in the peripheral blood and tissues of humanized mice were effectively depleted. Importantly, the size of the HIV reservoir was significantly decreased in anti-CD4 antibody treated animals following CD4^+^ T cell recovery compared with untreated controls.

## Introduction

Current antiretroviral therapy (ART) can effectively suppress HIV replication to undetectable levels. However, ART only inhibits new rounds of viral replication. It does not eradicate the HIV reservoir consisting of infected cells, a variable proportion of which harbor replication-competent, intact viral genomes that are not easily targetable for specific depletion by current approaches [1,2]. Withdrawal of ART inevitably leads to viremia rebound within a few weeks or months, necessitating lifelong therapy.

Induction of viral reservoir antigen expression with concomitant immune augmentation has emerged as a major strategy to deplete the reservoir [3–8]. While several viral reactivation strategies have been shown to be well-tolerated and resulted in increases in viral transcription in clinical trials, none has been shown to be effective in reducing the size of viral reservoir [3–8].

While further research may increase the efficacy of the viral antigen induction and clearance approach, a potential alternative strategy for reservoir depletion is to target host CD4^+^ T cells directly. Unlike anti-HIV broadly neutralizing antibodies (bNAbs) and other HIV-targeted immunotherapies in development, anti-CD4 antibody-mediated depletion of CD4^+^ T cells does not require the induction of viral antigen expression. Anti-CD4 antibody treatment has been used for treating various human diseases in clinical trials; in which the depletion of CD4^+^ T cells was reversible, well-tolerated, and generally with no clinical evidence of immunosuppression [9–12].

Anti-CD4 antibody-mediated CD4^+^ T cell depletion was used to study the role of CD4^+^ T cells in SIV infection and pathogenesis [13–16]. CD4^+^ T cell depletion enhanced infection of SIV in macrophages and microglia by shifting viral tropism [15]. CD4^+^ T cells contribute to post-peak decline of viremia in untreated SIV-infected rhesus macaques [16]. Antibody-mediated CD4^+^ T cell depletion induces homeostatic CD4^+^ T cell proliferation without detectable virus reactivation in ART-suppressed, SIV-infected macaques [14].

The results herein show that human CD4^+^ T cells are effectively depleted in both the peripheral blood and tissues of humanized mice by passive infusion of anti-human CD4 antibody. Importantly, the levels of CD4^+^ T cells recovered substantially upon discontinuation of antibody treatment. Significantly lower levels of cell-associated viral RNA and DNA were detected in anti-CD4 antibody-treated animals following reconstitution of CD4^+^ T cells as compared to controls. Notably, lower frequencies of intact proviral genomes likely to be replication-competent were present in the anti-CD4 antibody group relative to control following CD4^+^ T cell repopulation. In addition, a ~5-fold reduction in the number of latently infected cells harboring replication competent virus was noted. Taken together, the current study provides direct *in vivo* evidence of an effective approach to profoundly reduce the HIV reservoir.

## Results

### CD4^+^ T cell depletion by anti-CD4 antibodies

**Fig 1:**
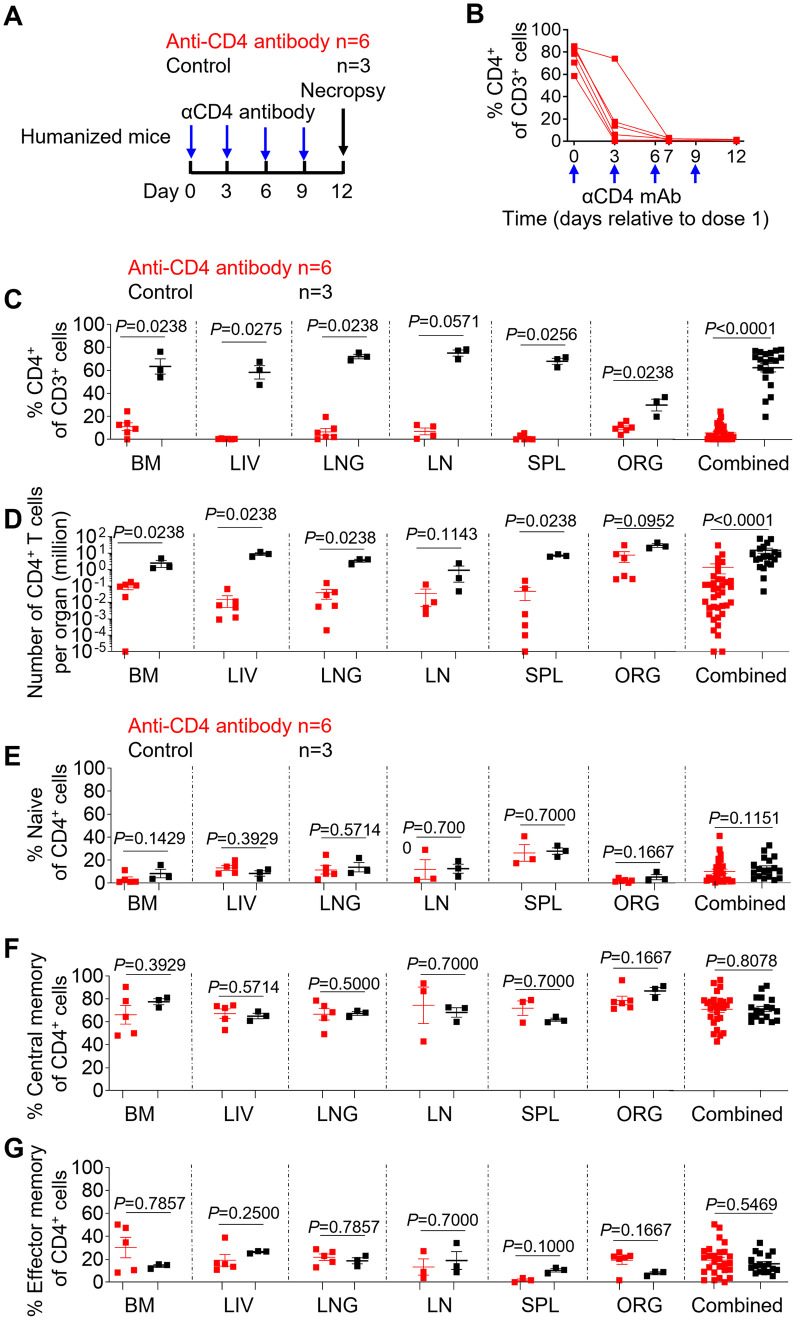
Human CD4^+^ T cells are robustly depleted in animals treated with anti-CD4 antibody. (A) Experimental design of anti-CD4 antibody treatment in humanized mice. (B) The frequency of human CD4^+^ T cells in peripheral blood was longitudinally monitored by flow cytometric analysis. The frequency (C) and the absolute number (D) of human CD4^+^ T cells in the tissues of humanized mice was determined by flow cytometric analysis at necropsy. The frequency of naïve (E, CD27^+^CD45RA^+^), central memory (F, CD27^+^CD45RA^-^), and effector memory (G, CD27^-^CD45RA^-^) CD4^+^ T cells from anti-CD4 antibody-treated and control animals was determined by flow cytometry at necropsy. BM, bone marrow; LIV, liver; LNG, lung; LN, lymph nodes; SPL, spleen, ORG, human thymic organoid. Combined: all individual tissues from all animals are graphed together. Blue arrows in A and B show the timing of 4 anti-CD4 antibody administrations (6 mg/kg) to the anti-CD4 antibody treated group. Anti-CD4 antibody-treated animals (n = 6) are shown in red; control animals (n = 3) are shown in black. Data are expressed as mean ± SEM. Statistical analyses were performed using unpaired two-sided Mann–Whitney U-tests. Statistical significance was considered when *P* < 0.05.

First, an experiment was designed to evaluate anti-CD4 antibody-mediated systemic depletion of CD4^+^ T cells in humanized mice (Fig 1A). For this purpose, we used an antibody that has been extensively used in previous *in vivo* experiments using NHP and that cross reacts with human CD4 [13–15]. Anti-CD4 antibody was administered to mice via intravenous injection at days 0, 3, 6, and 9. The levels of human CD4^+^ T cells were measured in the blood prior to and at days 3, 7, and 12 post antibody administration. The levels of human CD4^+^ T cells were measured in tissues at necropsy. The mean frequency of peripheral blood CD4^+^ T cells decreased by 75.5%, 98.0% and 98.9% at days 3, 7 and 12 after the first dose relative to the pre-depletion time point, respectively (Fig 1B). A robust depletion of CD4^+^ T cells in both percentages and absolute numbers was also observed in all analyzed tissues of antibody-treated animals (Fig 1C and 1D). In comparison to untreated controls, the mean frequency of CD4^+^ T cells in anti-CD4 antibody-treated animals decreased by 82.5% in the bone marrow (p = 0.0238), 99.3% in the liver (p = 0.0275), 90.8% in the lung (p = 0.0238), 90.8% in the lymph nodes (p = 0.0571), 97.8% in the spleen (p = 0.0256) and 66.2% in the human thymic organoid (p = 0.0238) and 90.9% when all individual tissues (p<0.0001) within the same group were analyzed together (Fig 1C). Similarly, the mean absolute number of CD4^+^ T cells in CD4-depleted animals decreased by 96.5% in the bone marrow (p = 0.0238), 99.8% in the liver (p = 0.0238), 98.9% in the lung (p = 0.0238), 96.2% in the lymph nodes (p = 0.1143), 99.3% in the spleen (p = 0.0238) and 74.9% in the human thymic organoid (p = 0.0952) and 91.0% when all individual tissues (p<0.0001) within the same group were analyzed together, respectively, in comparison to controls (Fig 1D).

Central memory CD4^+^ T cells are an important viral reservoir and have been reported to be more resistant to anti-CD4 antibody-mediated depletion than naïve or effector memory CD4^+^ T cells [13,17–19]. The phenotype of the remaining CD4^+^ T cells in tissues of humanized mice (CD27^+^CD45RA^+^ naïve, CD27^+^CD45RA^-^ central memory and CD27^-^CD45RA^-^ effector memory) were analyzed 3 days after the last dose of anti-CD4 antibody treatment. Despite the extensive CD4^+^ T cell depletion, no significant differences were observed in the proportion of each subset (naïve, Fig 1E, P = 0.1151; central memory, Fig 1F, P = 0.8078; effector memory, Fig 1G, p = 0.5469) between anti-CD4 antibody-treated and control mice suggesting that all subsets were well depleted by the antibody treatment. Collectively, these results demonstrate that CD4^+^ T cells can be efficiently depleted by anti-CD4 antibody treatment in humanized mice.

### Peripheral blood CD4^+^ T cells are effectively depleted in ART-suppressed, HIV-infected humanized mice receiving anti-CD4 antibodies and rapidly recover following cessation of antibody treatment

**Fig 2:**
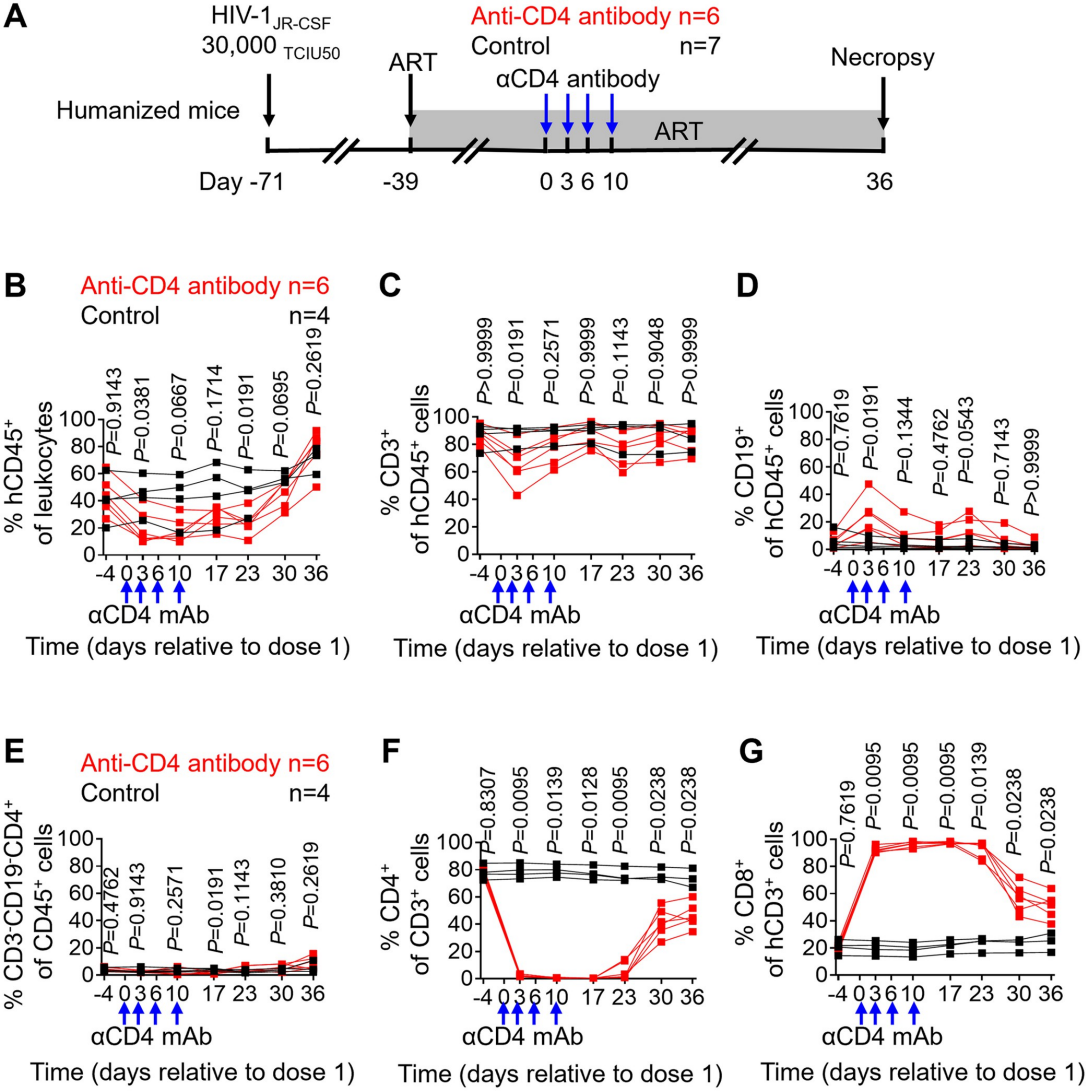
Peripheral blood CD4^+^ T cells are effectively depleted in ART-suppressed, HIV-infected humanized mice receiving anti-CD4 antibodies and rapidly recover following cessation of antibody treatment. (A) Experimental design of HIV infection and anti-CD4 antibody treatment in humanized mice. The frequency of human CD45^+^ hematopoietic cells (B), CD3^+^ T cells (C), CD19^+^ B cells (D), CD3^-^CD19^-^ myeloid cells (E), CD4^+^ T cells (F) and CD8^+^ T cells (G) in peripheral blood was longitudinally monitored by flow cytometric analysis. Blue arrows show the timing of 4 anti-CD4 antibody administrations (6 mg/kg) to the anti-CD4 antibody treatment group. Anti-CD4 antibody treated animals (n = 6) are shown in red; control animals (n = 7 in A; n = 4 in B-G) are shown in black. Data are expressed as mean ± SEM. Statistical analyses were performed using unpaired two-sided Mann–Whitney U-tests. Statistical significance was considered when *P* < 0.05.

To evaluate the effect of anti-CD4 antibody treatment on ART-suppressed, HIV-infected humanized mice, animals were intravenously infected with HIV at day -71 and treated with antiretroviral therapy at day -39, respectively (Fig 2A). Anti-CD4 antibody was administered at days 0, 3, 6 and 10. Following the final dose of anti-CD4 antibody, animals were maintained on suppressive ART for an additional 26 days to allow CD4^+^ T cell levels to recover.

The frequency of human cell subsets in the peripheral blood of mice was longitudinally monitored following the initiation of anti-CD4 antibody treatment. Significantly lower levels of human CD45^+^ cells and CD3^+^ T cells were observed in treated mice compared with controls at day 3 (CD45^+^ cells, p = 0.0381; CD3^+^ T cells, p = 0.0191) while no significant differences were observed at days 10, 17, 23, 30 and 36 (Fig 2B and 2C). In contrast, the frequency of CD19^+^ human B cell was significantly higher in anti-CD4 antibody-treated animals compared with control animals at day 3 (p = 0.0191) but not at days 10, 17, 23, 30 and 36 (Fig 2D).

In a previous study, the depletion of CD4^+^ T cells in ART-naïve SIV-infected macaques expanded macrophages and microglia and increased viral replication [15]. In contrast, a more recent study showed that monocytes were not depleted nor expanded in ART-suppressed SIV-infected macaques receiving anti-CD4 antibody treatment [13]. Therefore, the expansion of myeloid cells is very likely driven by active SIV replication. The frequency of human monocytes (identified as hCD45^+^hCD3^-^hCD19^-^hCD4^+^) in the peripheral blood of humanized mice was monitored longitudinally following the initiation of anti-CD4 antibody treatment. As shown in Fig 2E, no significant differences in human monocyte levels were observed over the course of anti-CD4 antibody treatment except on day 17 following the first dose of antibody (p = 0.0191) between anti-CD4 antibody-treated and control mice in peripheral blood.

Human CD4^+^ and CD8^+^ T cell levels in peripheral blood were also longitudinally measured following the first dose of anti-CD4 antibody treatment in ART-suppressed, HIV-infected animals. More than 95% of CD4^+^ T cells in peripheral blood were depleted over the course of antibody treatment (Fig 2F). CD4^+^ T cells in peripheral blood rebounded to 59.0% of pre-depletion levels by day 36 after antibody treatment initiation. The mean frequency of peripheral blood CD4^+^ T cells in anti-CD4 antibody-treated animals was reduced by 97.0%, 99.3%, 99.4%, 92.6%, 45.8% and 37.4% compared with controls at days 3 (p = 0.0095), 10 (p = 0.0139), 17 (0.0128), 23 (p = 0.0095), 30 (p = 0.0238) and 36 (p = 0.0238) post treatment initiation (Fig 2F). In contrast, the percentages of the peripheral blood CD8^+^ T cells in CD4-depleted animals rapidly increased following the first dose of anti-CD4 antibody administration and rapidly decreased after the final administration of antibody (Fig 2G). The mean frequency of CD8^+^ T cells in the anti-CD4 antibody treated group increased by 361.5%, 403.7%, 350.9%, 295.7%, 154.7% and 110.3% when compared to the control group at days 3 (p = 0.0095), 10 (p = 0.0095), 17 (p = 0.0095), 23 (p = 0.0139), 30 (p = 0.0238) and 36 (p = 0.0238) following the first antibody injection (Fig 2G).

CD8 depletion has been shown to promote CD4^+^ T cell activation and proliferation [20,21]. To examine the effect of CD4^+^ T cell depletion on T cell activation in the context of HIV infection and ART suppression, we next measured T cell activation (CD38^+^/HLA-DR^+^) levels during and following antibody treatment. No significant differences were found between anti-CD4 antibody-treated and control animals in the levels of activated CD4^+^ T cells throughout the course of the experiment (S1A Fig). Similarly, no significant differences were observed between anti-CD4 antibody-treated and control animals in the levels of activated CD8^+^ T cells in all time points, except on day 17 following antibody treatment initiation (p = 0.0191) (S1B Fig).

In a previous CD4 depletion study, central memory CD4^+^ T cells recovered faster than naïve CD4^+^ T cells, but all subsets returned to their pre-depletion levels 8–9 months post antibody therapy termination [13]. In the current study, higher levels of central memory (p = 0.0238) and effector memory CD4^+^ T cells (p = 0.0275) but lower levels of naïve CD4^+^ T cells (p = 0.0238) were observed in treated animals compared with controls at day 30 post anti-CD4 antibody treatment initiation (S2 Fig). However, no significant differences were found in the levels of naïve, central memory and effector memory cells one week later at the time of necropsy (S2 Fig). Together, these results demonstrate efficient recovery of human CD4^+^ T cells after antibody treatment termination.

### Anti-CD4 antibody administration reduces cell-associated viral RNA and DNA levels in peripheral blood

**Fig 3:**
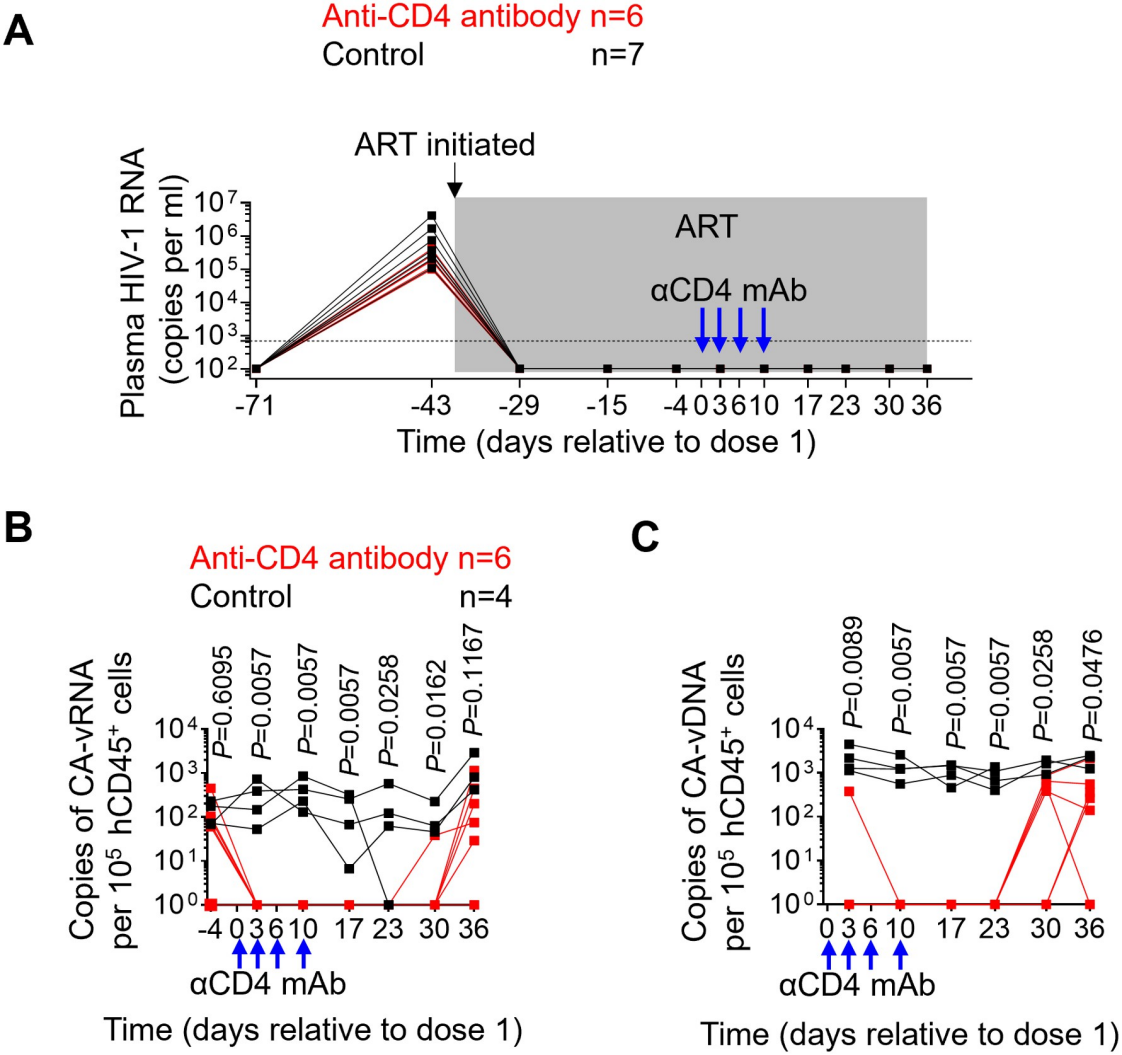
Anti-CD4 antibody administration reduces cell-associated viral RNA and DNA levels in peripheral blood. (A) Plasma viral load (HIV-RNA copies/ml) was quantified in longitudinal plasma samples using a qRT-PCR assay following HIV-1 infection. (B) Levels of cell-associated viral RNA in PBMCs were longitudinally quantified by qPCR. (C) Levels of cell-associated viral DNA in PBMCs were longitudinally quantified by qPCR. PBMC: peripheral blood mononuclear cell. The shaded gray area in A represents ongoing ART administration. The dotted line in panel A indicates the limit of detection (693 copies/ml). Samples in B and C with undetectable values are set as 1 copy per 10^5^ hCD45^+^ cells. Blue arrows show the timing of 4 anti-CD4 antibody administrations (6 mg/kg) to the anti-CD4 antibody treatment group. Anti-CD4 antibody treated animals (n = 6) are shown in red; control animals (n = 7 in A; n = 4 in B and C) are shown in black. Data are expressed as mean ± SEM. Statistical analyses were performed using unpaired two-sided Mann–Whitney U-tests. Statistical significance was considered when *P* < 0.05.

Plasma viral load was longitudinally monitored following HIV exposure. Treatment of ART-suppressed, HIV-infected mice with anti-CD4 antibody did not result in an induction of plasma viremia (Fig 3A). This is in contrast to previously published results using CD8-depleting antibodies [20,22]. Cell-associated viral RNA levels following the initiation of anti-CD4 antibody treatment were also monitored longitudinally. Cell-associated viral RNA levels were significantly lower in animals treated with anti-CD4 depleting antibody 3 (p = 0.0057), 10 (p = 0.0057), 17 (p = 0.0057), 23 (p = 0.0258) and 30 (p = 0.0162) days following the first dose of antibody treatment as compared to the untreated control group (Fig 3B). Mean cell-associated viral RNA levels were 3.4-fold lower in anti-CD4 antibody treated animals compared with controls at day 36, although the difference was not statistically significant (Fig 3B; p = 0.1167).

Longitudinal analysis of cell-associated vial DNA levels in peripheral blood hCD45^+^ cells between anti-CD4 antibody treated and untreated control mice was also performed. Cell-associated viral DNA levels were significantly lower 3 (p = 0.0089), 10 (p = 0.0057), 17 (p = 0.0057), 23 (p = 0.0057), 30 (p = 0.0258) and 36 (p = 0.0476) days after the first dose of antibody in anti-CD4 antibody-treated animals compared with untreated controls (Fig 3C). Collectively, these data indicate that depletion of CD4^+^ T cells results in significant decreases in the levels of cell-associated viral RNA and DNA levels in peripheral blood.

### CD4^+^ T cell depletion decreases the levels of HIV RNA and DNA in CD4^+^ T cells

**Fig 4:**
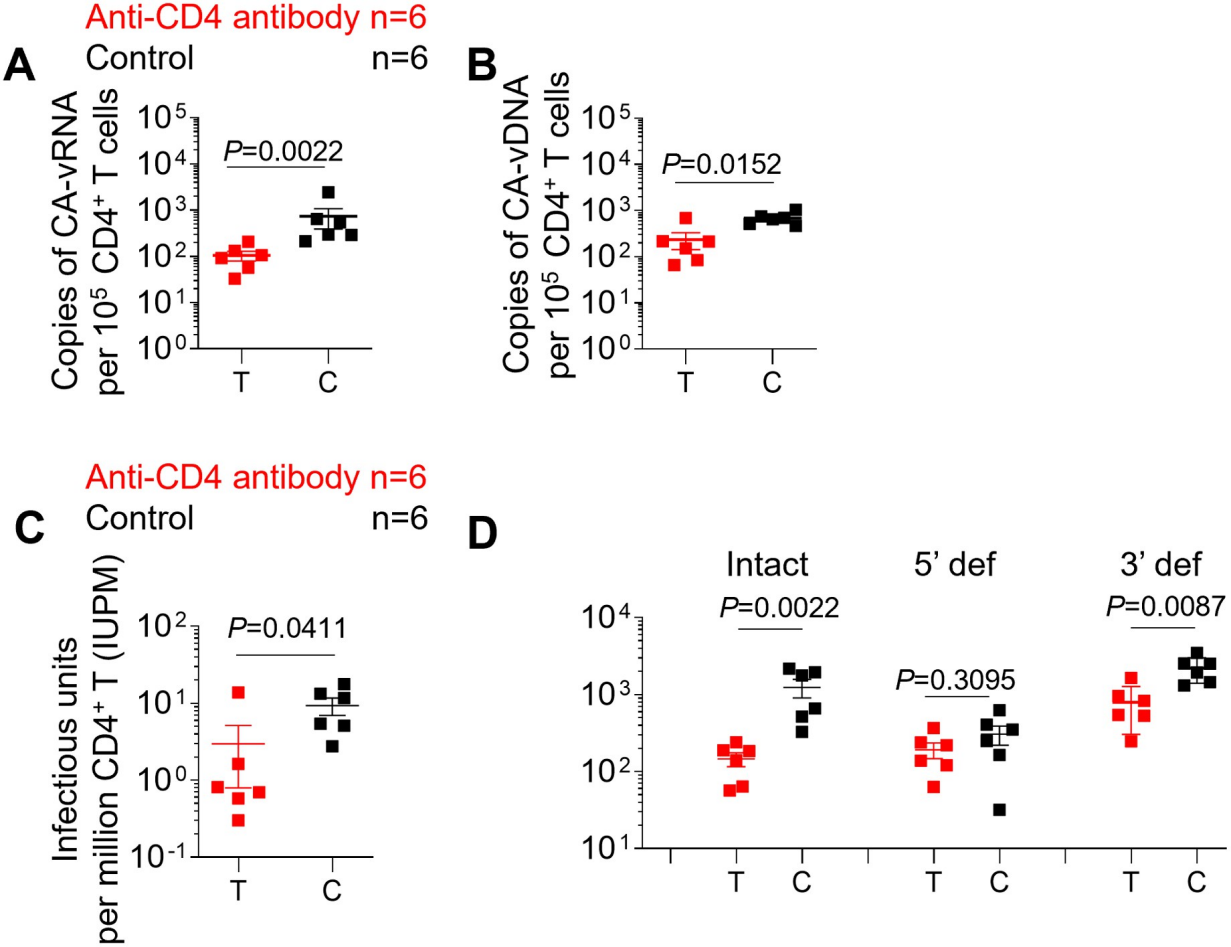
CD4^+^ T cell depletion results in a reduction in the levels of intact HIV proviruses and latently infected cells. Copies of cell-associated viral RNA (A) and DNA (B) levels in purified CD4^+^ T cells isolated from pooled MNCs were quantified by qPCR. (C) Copies of intact virus per million purified CD4^+^ T cells isolated from pooled MNCs were measured by quantitative viral outgrowth assay (QVOA). (D) The frequency of intact virus, 5’ defective virus and 3’ defective virus in purified CD4^+^ T cells isolated from pooled MNCs was measured by the intact proviral DNA assay (IPDA). MNC: mononuclear cell; Intact, intact virus; 5’ def, 5’ defective virus; 3’ def, 3’ defective virus. Anti-CD4 antibody treated animals (n = 6) are shown in red; control animals (n = 6) are shown in black. T and C in the X axis represent Test and Control, respectively. Data are expressed as mean ± SEM. Statistical analyses were performed using unpaired two-sided Mann–Whitney U-tests. Statistical significance was considered when *P* < 0.05.

To determine the effect of antibody-mediated CD4^+^ T cell depletion on cell-associated viral RNA and DNA levels in the tissues of ART-suppressed HIV-infected humanized mice following CD4^+^ T cell repopulation, human CD4^+^ T cells were enriched from pooled mononuclear cells (MNCs) isolated from the bone marrow, liver, lung, spleen, lymph nodes and human thymic organoid of each mouse. First, the effect of CD4^+^ T cell depletion on cell-associated viral RNA in purified CD4^+^ T cells was evaluated. Significantly lower levels of cell-associated viral RNA were detected in CD4^+^ T cells isolated from anti-CD4 antibody-treated animals compared with control animals (Fig 4A; P = 0.0022). Next, we examined the effect of CD4^+^ T cell depletion on cell-associated viral DNA levels. Substantially lower levels of cell-associated viral DNA were detected in CD4^+^ T cells isolated from anti-CD4 antibody-treated animals compared with control animals (Fig 4B; P = 0.0152).

### CD4^+^ T cell depletion results in a reduction in the levels of intact HIV proviruses and latently infected cells

To determine whether the observed declines in cell-associated viral RNA and DNA were reflected in the level of intact HIV proviruses or the number of latently infected cells, we performed a quantitative viral outgrowth assay (QVOA) and an intact proviral DNA assay (IPDA). There was a mean 3.1-fold decrease in the number of latently infected cells per million CD4^+^ T cells in anti-CD4 antibody-treated animals compared with controls (p = 0411) (Fig 4C). One animal did not seem to respond to treatment. When the 5 animals that responded to treatment were compared with the controls, the number of latently infected CD4^+^ T cells was reduced 11.5-fold (Fig 4C). Importantly, the mean frequency of intact proviruses measured by IPDA was decreased 8.5-fold in anti-CD4 antibody-treated mice compared with control mice and this difference was statistically significant (P = 0.0022) (Fig 4D). The frequency of 3’ defective proviruses was also significantly lower in anti-CD4 antibody-treated mice compared with control mice (Fig 4D; p = 0.0087). No significant difference was observed in the frequency of 5’ defective proviruses between anti-CD4 antibody-treated and control mice (Fig 4D; p = 0.3095). Collectively, these results demonstrate that antibody-mediated depletion of CD4^+^ T cells results in a decrease in the size of the HIV-1 reservoir.

### Systemic recovery of CD4^+^ T cell levels after antibody treatment termination in ART-suppressed, HIV-infected mice

**Fig 5:**
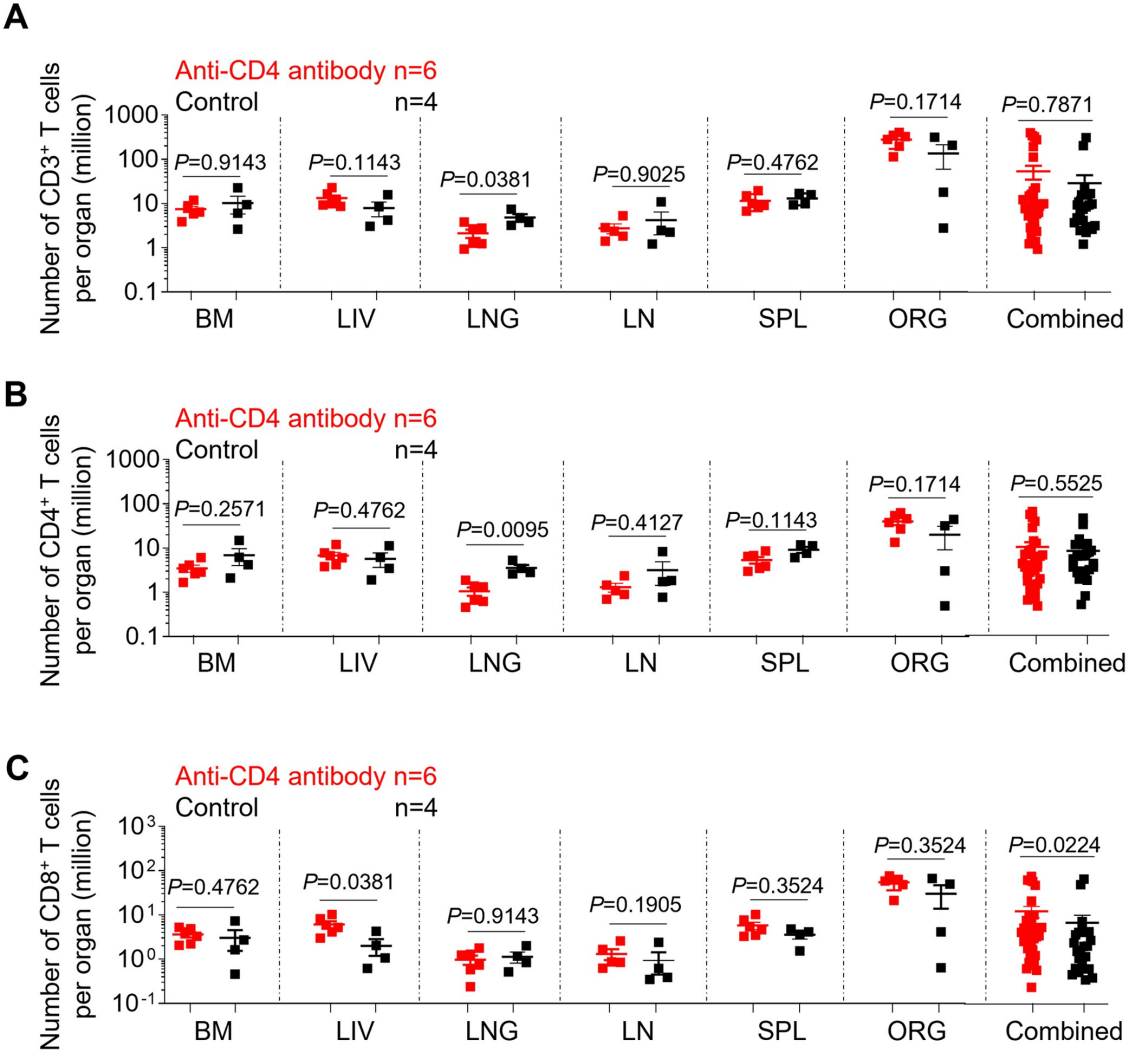
Systemic recovery of CD4^+^ T cell levels after antibody treatment termination in ART-suppressed, HIV-infected mice. The absolute number of human CD3^+^ T cells (A), CD4^+^ T cells (B) and CD8^+^ T cells (C) in the tissues of anti-CD4 antibody-treated and control animals was determined by flow cytometry at necropsy. BM, bone marrow; LIV, liver; LNG, lung; LN, lymph nodes; SPL, spleen, ORG, human thymic organoid. Combined: all individual tissues from all animals are graphed together. Anti-CD4 antibody treated animals (n = 6) are shown in red; control animals (n = 4) are shown in black. Data are expressed as mean ± SEM. Statistical analyses were performed using unpaired two-sided Mann–Whitney U-tests. Statistical significance was considered when *P* < 0.05.

Systemic human CD3^+^ T cell levels were compared between anti-CD4 antibody-treated and control animals at necropsy. There was no significant difference in the frequency of human CD3^+^ T cells in all tissues tested, except for the liver, between the anti-CD4 antibody-treated and the control groups (p = 0.0381) (S3A Fig). Similarly, no significant difference was observed in the absolute number of CD3^+^ T cells in all tissues, except for the lung, between anti-CD4-antibody-treated and control animals (p = 0.0381) (Fig 5A). Overall, no significant differences were found in the frequency or absolute number of CD3^+^ T cells between the test group and the control group (Figs S3A and 5A).

A comparison of CD4^+^ T cell levels in the tissues of anti-CD4 antibody-treated and control mice following reconstitution was also performed. Interestingly, although the frequency of human CD4^+^ T cells was significantly lower in the bone marrow (p = 0.0139), liver (p = 0.0191), lung (p = 0.0247), lymph nodes (p = 0.0159) and spleen (p = 0.0191) of anti-CD4 antibody-treated compared with control mice, a statistically significant difference in the absolute numbers of CD4^+^ T cells was only observed in the lung (p = 0.0095) (Figs S3B and 5B).

Next, human CD8^+^ T cell levels in tissues were compared between anti-CD4 antibody-treated mice and controls. The percentages of CD8^+^ T cells in the bone marrow (p = 0.0139), liver (p = 0.0191), lung (p = 0.0247), lymph nodes (p = 0.0159) and spleen (p = 0.0191) of anti-CD4 antibody-treated mice were significantly higher as compared to control mice (S3C Fig). However, no significant differences were found in the absolute number of CD8^+^ T cells in all individual tissues analyzed, except for the lung, between anti-CD4 antibody-treated and control mice (p = 0.0381) (Fig 5C). Together these results indicate that even though there was a significant recovery of the overall levels of CD4^+^ T cells at day 26 following antibody treatment interruption, the CD4^+^ T cell compartment did not fully return to the same levels observed in control animals.

T cell activation levels in the tissues of anti-CD4 antibody-treated and control mice at necropsy were also compared. No significant differences were observed in the levels of CD4^+^ or CD8^+^ T cell activation in any of the individual tissues analyzed (S4 Fig).

Next, we measured the proportion of different CD4^+^ T cell subsets in tissues. Significantly lower levels of naïve (p = 0.0191) CD4^+^ T cells were noted, whereas higher levels of central memory (p = 0.0191) and effector memory (p = 0.0191) CD4^+^ T cells were observed in the bone marrow of anti-CD4 antibody-treated animals as compared to controls (S5 Fig). Similarly, lower levels of naïve CD4^+^ T cells and higher levels of central memory CD4^+^ T cells were also observed in the lung of anti-CD4 antibody-treated mice (S5 Fig). Overall, significantly lower levels of naïve CD4^+^ T cells (p = 0.0002) and significantly higher levels of central memory CD4^+^ T cells (p = 0.0002) were observed in anti-CD4 antibody treated animals (S5 Fig).

### CD4^+^ T cell depletion does not delay viral rebound after ATI

In a separate experiment, we tested whether the reduction in latently infected cells and intact proviruses observed following anti-CD4 antibody treatment would be reflected in a delay in viral rebound after analytical ART interruption (S6A Fig). Robust depletion of CD4^+^ T cells in peripheral blood was achieved throughout the course of anti-CD4 antibody treatment (S6B and S6C Fig). Compared with pre-depletion levels, the mean frequency of peripheral blood CD4^+^ T cells in anti-CD4 antibody-treated animals reduced by 96.8%, 99.3%, 99.3%, 99.5%, respectively, at days 2, 9, 17, 23 following the first dose of antibody. The frequency of the peripheral blood CD4^+^ T cells began to rebound 16 days after the last dose and recovered to 59.9% of pre-depletion levels before ART interruption. However, after ART interruption, no delay in viral rebound was observed in anti-CD4 antibody-treated animals (S6D Fig).

## Discussion

CD4^+^ T cells represent the most well-characterized and largest HIV reservoir. No specific cellular markers are currently available to reliably discriminate latently infected cells from uninfected ones [1,2]. Although HIV latency reversal agents are capable of inducing expression of RNA in clinical trials, none have been shown to successfully reduce the viral reservoir [3–8]. Broadly neutralizing antibodies (bNAbs), bi-specific antibodies, and toxin-tagged antibodies have the potential to kill HIV-1-infected cells, but their effects are highly dependent on HIV reactivation. Alternatives are needed that are not based on the targeting of HIV infected cells expressing HIV antigens. Importantly, latently infected CD4^+^ T cells are highly heterogeneous making their elimination difficult. Clinical proof-of-concept exists for passive administration of antibodies to deplete specific cell types across several disease areas [22–24]. Specifically, T-cell depletion with anti-thymocyte globulin has a long history of use for the treatment of graft versus host disease, transplant conditioning, transplant rejection, and several autoimmune diseases [25–27]. Notably, muromonab (anti-CD3), the first FDA-approved monoclonal antibody, was historically used for anti-kidney transplant rejection. However, pan T-cell depletion treatment would also deplete CD8^+^ T cells that have a critical role in controlling HIV and numerous other infections. Additionally, pan T-cell depleting therapies are associated with a cytokine release syndrome and other toxicities [28]. In contrast, CD4-T cell depleting antibodies have the significant advantage to spare CD8 T cells.

In previous clinical studies CD4 targeted antibodies were evaluated for the potential treatment of various human diseases [9–12]. Treatment with anti CD4 antibodies showed improvements in clinical and laboratory parameters. Importantly, only occasional and minor sides effects were reported. Previous studies performed using NHP also indicated that anti-CD4 antibody mediated CD4^+^ T cell depletion is reversible and well tolerated [13,14].

Anti-CD4 antibodies have been shown to deplete CD4^+^ T cells in previous non-human primate studies [13–16]. In the current study, an anti-CD4 antibody was administered to target all human CD4^+^ T cells regardless of HIV status. CD4^+^ T cells were efficiently depleted by the anti-CD4 antibody in peripheral blood and tissues. Specifically, we show that naïve, central memory and effector memory human CD4^+^ T cells were proportionally depleted in all tissues analyzed.

Consistent with a previous non-human primate study, anti-CD4 antibody treatment did not expand monocytes in the peripheral blood of ART-suppressed HIV-infected humanized mice [13]. In another study, anti-CD4 antibody was administered before SIV infection [15]. Monocytes and microglia were activated and expanded after SIV infection rather than during anti-CD4 antibody administration or following ART initiation [15]. Therefore, the expansion of myeloid cells is very likely driven by active SIV replication. Furthermore, the T-tropic HIV-1_JR-CSF_ used in the current study does not replicate in macrophages [29]. Of note, significantly lower levels of monocytes were observed in antibody-treated animals as compared to controls at day 17 following the first dose of antibody. This is probably due to the partial depletion of monocytes, which express low levels of CD4 molecules on their surface.

CD4^+^ T cell depletion did not induce T cell activation in humanized mice or plasma viral blips. In contrast, antibody-mediated depletion of CD8^+^ cells induce CD4^+^ T cell activation, drives a transient proliferative expansion of CD4^+^ T cells and induces HIV from latency in rhesus macaques [20–22].

CD4^+^ T cell depletion resulted in significant reductions in cell-associated viral RNA and DNA in the peripheral blood human CD45^+^ cells over the course of anti-CD4 antibody treatment. Similar to our results, lower levels of cell-associated SIV DNA were detected in PBMCs following anti-CD4 antibody treatment in macaques [14]. Furthermore, significantly lower levels of cell-associated viral RNA and DNA were detected in tissues of anti-CD4 antibody-treated animals following CD4^+^ T cell recovery compared with control animals, perhaps because of the replacement of HIV-infected cells with the newly reconstituted CD4^+^ T cells protected from HIV infection by continuous ART.

A 8.5-fold reduction was achieved in the levels of intact proviral DNA in tissues of anti-CD4-antibody-treated humanized mice in comparison to controls. In addition, in the majority of animals examined, we observed a 11.5-fold reduction in the number of latently infected cells in tissues. Consistent with our results, a recent study in ART suppressed, SIV-infected NHPs also reported a 0.5 log reduction in the number of latently infected cells in peripheral blood after CD4^+^ T cell depletion. However this reduction was not statistically significant [14].

The lack of statistically significant depletion of 5’-defective proviruses is unlikely to be explained by preferential depletion of 3’-defective and/or intact proviruses, as the anti-CD4 antibody should be agnostic to proviral intactness in the context of suppressive ART. The 2 amplicons in the IPDA that together exclude defective proviruses were designed based on near-full length proviral sequences from humans on years of durable ART who initiated treatment mainly during chronic infection. Given the differences in the duration of treatment in humans versus in mice, it is difficult to know how well the IPDA amplicons exclude defective proviruses in the humanized mouse samples because the proviral deletion/hypermutation landscape is likely different from humans treated during chronic HIV infection on long-term ART (though it is difficult to know with certainty without sequencing and comparing). Another consideration is that the predictive value of the IPDA primers/probe for intact proviruses appears to vary across different human donors due to inter-individual proviral landscape variability [30], and therefore likely also varies across individual humanized mice. Additionally, the small sample size of this study could amplify small differences across individual mice. All of these could potentially contribute to the lack of decrease in 5’-defective provirus.

An inverse expansion of CD8^+^ T cells follows the depletion of CD4^+^ T cell mediated by anti-CD4 antibody in rhesus macaques [13]. In line with the previous study, an expansion of CD8^+^ T cells was also observed in the current study, perhaps due to the incomplete recovery of CD4^+^ T cells. Anti-HIV-1-specific CD8^+^ T cell responses in humanized mice are similar to those in humans in terms of their specificity, kinetics, and immunodominance [31]. Anti-HIV-specific CD8^+^ T cell responses following HIV infection have been demonstrated to be important in controlling viral replication in humanized mice [31]. More recently, a moderate level of virus reactivation has been observed in ART-suppressed humanized mice after CD8 depletion [22]. Therefore, increased cytotoxic CD8^+^ T cell responses against HIV reservoir cells actively expressing HIV antigens may also contribute to observed decreases in HIV parameters in the current study. While a significantly lower frequency of CD4^+^ T cells was observed in anti-CD4 antibody treated animals at necropsy, no difference in the absolute numbers of CD4^+^ T cells was observed, which may be partially due to the expansion of CD8^+^ T cells.

Central memory and effector memory CD4^+^ cells recovered faster than naïve CD4^+^ T cells in the peripheral blood of anti-CD4 antibody treated animals, but eventually all returned to levels similar to those in the control animals. This phenomenon is consistent with previous observations in non-human primates [13]. Higher percentages of memory CD4^+^ T cells while lower levels of naïve CD4^+^ T cells were found in anti-CD4 antibody-treated animals at necropsy. Further studies will be required to determine if this difference resolves over time and if not, what is its functional relevance. The relatively small numbers of CD4^+^ T cells in the current study prevented the investigation of the contribution of different subsets of CD4^+^ T cells to the HIV reservoir.

There are some limitations of the use of humanized mice in HIV/AIDS research. The reconstituted human immune system in mice partially recapitulates the human immune system. The limited generation of anti-HIV IgG antibodies probably underestimates the impact of anti-antibodies, which is one concern of antibody-mediated clinical interventions. In addition, the relatively small cell numbers in peripheral blood or tissues of humanized mice when compared to humans and NHPs precludes the full analysis of the HIV reservoir on specific cell subsets.

Despite the significant reduction in the number of intact proviruses present in the treated animals and the reduction in latently infected cells containing replication competent HIV, there was no observable delay in viral rebound upon ART interruption. This is consistent with previous work in non-human primate where depletion of CD4^+^ T cells did not delay viral rebound [13]. Therefore, despite these highly promising results further refinement, longer treatment, or the use of combination therapies will be needed to delay viral rebound after ATI. In summary, our results provide direct *in vivo* evidence that transient depletion of CD4^+^ T cells is a viable approach to efficiently reduce the size of the HIV reservoir that is agnostic to the HIV infection status of cells.

## Materials and methods

### Ethics statement

All animal experiments were conducted according to protocols approved by the Institutional Use and Care Committee at the University of North Carolina-Chapel Hill and in compliance with the National Institutes of Health Guide for the Care and Use of Laboratory Animals.

### Generation of humanized mice

Humanized mice were generated as previously described [22,32–38]. In brief, one small piece of human fetal liver tissue (1–2 mm) was sandwiched between two pieces of autologous thymus tissue (Advanced Bioscience Resources) under the left kidney capsule of sublethally irradiated (200 rad) NOD.Cg-*Prkdc*^*scid*^*Il2rg*^*tm1Wjl*^/SzJ (NSG; The Jackson Laboratory) mice. Following tissue implantation, mice were transplanted intravenously (via tail vein injection) with autologous liver derived human CD34^+^ hematopoietic stem cells. Reconstitution of BLT mice with human hematopoietic cells in peripheral blood was monitored longitudinally by flow cytometry as previously described [22,32–38].

### HIV-1 infection of humanized mice

Stocks of HIV-1_JR-CSF_ were prepared and titrated as previously reported [34,37,38]. Briefly, viral-containing supernatants were produced via transient transfection of HEK293T cells and were titrated on TZM-bl indicator cells (AIDS Research and Reference Reagent Program, Division of AIDS, National Institute of Allergy and Infectious Diseases). Humanized mice were intravenously (via tail vein) exposed to 3 × 10^4^ tissue culture infectious units (TCIU) of HIV_JR-CSF_.

### Antiretroviral treatment and anti-CD4 antibody administration

ART was administered to humanized mice using irradiated Teklad chow diet containing the following three drugs: emtricitabine (FTC; 1,500 mg per kg), tenofovir disoproxil fumarate (TDF; 1,560 mg per kg) and raltegravir (RAL; 600 mg per kg) (Research Diets) as previously described [22,34,37,38].

For anti-CD4 antibody administration, each animal was intravenously administered with four doses of rhesusized depleting anti-CD4 antibody (CD4R1, NIH Nonhuman Primate Reagent Resource) at a dose of 6 mg per kg. The antibody was diluted in saline and intravenously administered (via tail vein) every 3 to 4 days.

### Mononuclear cell isolation

Mononuclear cells (MNCs) were isolated from tissues as previously described [35,38–40]. Briefly, mononuclear cells from the spleen, lymph nodes and human thymic organoid were obtained by passing the tissue through a 70 μm nylon cell strainer with a 3 ml syringe plunger. Bones were pulverized with a mortar and pestle before passage through a cell strainer. Lung and liver tissues were minced into small pieces and digested with collagenase /DNase prior to filtering tissue through a cell strainer. The cells were further purified by centrifugation through a Percoll gradient. Tissue red blood cells were lysed with ACK lysis buffer.

### Human CD4^+^ T cell purification

All mononuclear cells isolated from the bone marrow, liver, lung, spleen, lymph nodes, and 20 million mononuclear cells isolated from human thymic organoid of each animal were pooled together before proceeding with CD4^+^ T cell selection. CD4^+^ T cells from each pooled sample were positively enriched by anti-human CD4 microbeads (Miltenyi Biotec kit 130-045-101). Flow cytometric analysis was carried out pre and post selection to assess the purity of the sorted samples.

### Plasma viral load, cell-associated HIV-1 RNA and DNA

RNA was extracted from plasma samples using the Qiagen RNeasy Mini kit. Blood and tissue RNA samples were extracted using Qiagen QIAamp viral RNA Mini Kit. Plasma HIV viral loads were monitored longitudinally with a sensitivity of 693 copies per ml. Viral RNA was quantified with a one-step reverse-transcriptase qPCR using TaqMan RNA to-CT 1-step kit, which was performed on an ABI 7500 Fast Real-Time PCR System (Applied Biosystems) as previously described [22,34,38]. The sequences of the forward and reverse primers and the TaqMan probe were: 5′-CATGTTTTCAGCATTATCAGAAGGA-3′, 5′-TGCTTGATGTCCCCCCACT-3′ and 5′-FAM-CCACCCCACAAGATTTAAACACCAT-GCTAA-Q-3′, respectively.

DNA samples were isolated using Qiagen QIAamp DNA Blood Mini Kit. Copies of cell-associated viral DNA in peripheral blood and tissues were quantified by qPCR analysis as previously described [32,38,39]. Human cell numbers were determined by simultaneously amplifying human gamma globin DNA. The sequences for the forward and reverse primers and probe for the detection of human gamma globin were 5’-CGCTTCTGGAACGTCTGAGATT-3’, 5’-CCTTGTCCTCCTCTGTGAAATGA-3’ and 5’-FAM-TCAATAAGCTCCTAGTCCAGAC-Q-3’, respectively.

### Flow cytometry analyses

Human immune cells from humanized mice were analyzed by a 9-color flow cytometry panel, which included CD45-V500 (HI30, BD Biosciences), CD3-APC-R700 (UCHT1, BD Biosciences), CD4-APC-H7 (RPA-T4, BD Biosciences), CD8-FITC (SK1, BD Biosciences), CD19-PE-Cy7 (SJ25C1, BD Biosciences), CD27-PE (M-T271, BD Biosciences), CD38-APC (HB7, BD Biosciences), CD45RA-Pacific Blue (F8-11-13, Bio-Rad), HLA-DR-PerCP (L243, BD Biosciences). Mouse IgG1k-APC (MOPC-21, BD Biosciences), mouse IgG1k- Pacific Blue (MOPC-21, BD Biosciences), mouse IgG1k-PE (MOPC-21, BD Biosciences) and mouse IgG2ak-PerCP (X39, BD Biosciences) were used as isotype controls. Blood samples were lysed with 1× BD FACS lysing solution (BD Bio-sciences) following antibody incubation. Samples were then washed and fixed with PFA. Data was collected on a BD LSRFortessa instrument and analyzed with BD FACSDiva (version 6.1.3) and FlowJo (version 10.6.2) software.

Flow cytometric gating strategies were as follows: hCD45^+^hCD3^+^ and hCD45^+^CD19^+^ were used to gate human T cells and B cells, respectively; co-expression of human CD38 and HLA-DR were characterized as activated T cells; T cell subsets were gated based on the expression of CD27 and CD45RA (naïve: CD27^+^CD45RA^+^, central memory: CD27^+^CD45RA^-^, effector memory: CD27^-^CD45RA^-^); CD3^-^CD19^-^CD4^+^ were used to gate human monocytes (S7 Fig).

### Intact proviral DNA assay

IPDA was performed on isolated total CD4^+^ T cells as described [41]. A median of 4.2 (Q1 3.2, Q3 7.8) x 10^5^ CD4^+^ T cell equivalents were assayed per sample. Median DNA shearing index was 0.47 (Q1 0.41, Q3 0.49). Proviral frequencies less than 5 x 10^6^ CD4^+^ T cells were left-censored. Digital PCR thresholds for positive proviral amplification were set using no template, HIV seronegative human donor CD4^+^ T cell DNA, and HIV amplicon gblock (Integrated DNA Technologies) controls.

### Quantitative viral outgrowth assay

The QVOA was performed as previously described. Briefly, human CD4^+^ T cells were purified from mononuclear cells as reported [21]. CD4^+^ T cells were plated in limiting dilution and stimulated for 24 hours with a cocktail consisting of 2ug/ml PHA (Remel, Fisher Scientific), 60U Il-2 and irradiated PBMC from an uninfected human donor. CD8-depleted, PHA blasts generated from PBMC of seronegative human donors were added to cultures to expand reactivated virus. The frequency of CD4^+^ T cells containing replication competent HIV was estimated using a maximum likelihood approach [21].

### Statistical analyses

All data were graphed and analyzed using GraphPad Prism (version 8.02). Data are expressed as mean ± SEM. Statistical significance was considered when p < 0.05. No statistical methods were used to predetermine sample size. Investigators were not blinded to group allocations or when assessing outcomes. To assess the statistical significance of the differences observed between anti-CD4 antibody group and the control group, we used unpaired two-sided Mann–Whitney U-tests.

## Supporting information

S1 Fig Anti-CD4 antibody treatment does not induce T cell activation in blood. The frequency of activated (CD38^+^HLA-DR^+^) CD4^+^ (A) and CD8^+^ (B) T cells in the peripheral blood was longitudinally determined by flow cytometry. Blue arrows in A and B show the timing of 4 anti-CD4 antibody administrations to anti-CD4 antibody treated animals. Anti-CD4 antibody treated animals (n = 6) are shown in red; control animals (n = 4) are shown in black. Data are expressed as mean ± SEM. Statistical analyses were performed using unpaired two-sided Mann–Whitney U-tests. Statistical significance was considered when *P* < 0.05.
(TIF)

S2 Fig Memory CD4^+^ T cells repopulated faster than naïve CD4^+^ T cells in blood following termination of antibody treatment. The frequency of naïve (A, CD27^+^CD45RA^+^), central memory (B, CD27^+^CD45RA^-^), and effector memory (C, CD27^-^CD45RA^-^) CD4^+^ T cells in the peripheral blood was longitudinally determined by flow cytometry. Blue arrows in A show the timing of 4 anti-CD4 antibody administrations to the anti-CD4 antibody treatment group. Anti-CD4 antibody treated animals (n = 6) are shown in red; control animals (n = 4) are shown in black. Data are expressed as mean ± SEM. Statistical analyses were performed using unpaired two-sided Mann–Whitney U-tests. Statistical significance was considered when *P* < 0.05.
(TIF)

S3 Fig Significantly lower percentage of CD4^+^ T cells is observed in ART-suppressed, HIV-infected animals receiving anti-CD4 antibody treatment compared with controls following CD4^+^ T cell recovery. The frequency of human CD3^+^ T cells (A), CD4^+^ T cells (B) and CD8^+^ T cells (C) in tissues of anti-CD4 antibody-treated and control animals was determined by flow cytometry at necropsy. BM, bone marrow; LIV, liver; LNG, lung; LN, lymph nodes; SPL, spleen, ORG, human thymic organoid. Combined: all individual tissues from all animals are graphed together. Anti-CD4 antibody treated animals (n = 6) are shown in red; control animals (n = 4) are shown in black. Data are expressed as mean ± SEM. Statistical analyses were performed using unpaired two-sided Mann–Whitney U-tests. Statistical significance was considered when *P* < 0.05.
(TIF)

S4 Fig Anti-CD4 antibody treatment does not induce T cell activation in the tissues of ART-suppressed, HIV-infected humanized mice following CD4^+^ T cell reconstitution. The frequency of activated (CD38^+^HLA-DR^+^) CD4^+^ (A) and CD8^+^ (B) T cells was determined by flow cytometry at necropsy. BM, bone marrow; LIV, liver; LNG, lung; LN, lymph nodes; SPL, spleen, ORG, human thymic organoid. Combined: all individual tissues from all animals are graphed together. Anti-CD4 antibody treated animals (n = 6) are shown in red; control animals (n = 4) are shown in black. Data are expressed as mean ± SEM. Statistical analyses were performed using unpaired two-sided Mann–Whitney U-tests. Statistical significance was considered when *P* < 0.05.
(TIF)

S5 Fig Higher levels of central memory CD4^+^ T cells are detected in anti-CD4 antibody treated animals following reconstitution. The frequency of naïve (A, CD27^+^CD45RA^+^), central memory (B, CD27^+^CD45RA^-^), and effector memory (C, CD27^-^CD45RA^-^) CD4^+^ T cells from anti-CD4 antibody-treated and control animals was determined by flow cytometry at necropsy. BM, bone marrow; LIV, liver; LNG, lung; LN, lymph nodes; SPL, spleen, ORG, human thymic organoid. Combined: all individual tissues from all animals are graphed together. Anti-CD4 antibody treated animals (n = 6) are shown in red; control animals (n = 4) are shown in black. Data are expressed as mean ± SEM. Statistical analyses were performed using unpaired two-sided Mann–Whitney U-tests. Statistical significance was considered when *P* < 0.05.
(TIF)

S6 Fig Anti-CD4 antibody treatment does not delay viral rebound following ART interruption. (A) Experimental design of HIV infection and anti-CD4 antibody treatment in humanized mice. (B) The frequency of CD4^+^ T cells in the peripheral blood was longitudinally monitored by flow cytometric analysis. (C) Flow plots showing the fraction of CD4^+^ T cells in anti-CD4 antibody-treated (top) and control (bottom) mice. (D) Plasma viral load (HIV-RNA copies/ml) was quantified in longitudinal plasma samples using a qRT-PCR assay following HIV-1 infection. Blue arrows show the timing of 5 anti-CD4 antibody administrations (6 mg/kg) to the anti-CD4 antibody treatment group. Anti-CD4 antibody treated animals (n = 5) are shown in red; control animals (n = 1) are shown in black.
(TIF)

S7 Fig Gating strategies for flow cytometry. Shown are gating strategies for the analysis of (A), human immune cell subsets (hCD45^+^CD3^+^: T cells, hCD45^+^CD19^+^: B cells); (B), T cell activation (CD38^+^HLA-DR^+^); (C), T cell subsets (naïve: CD27^+^CD45RA^+^, central memory: CD27^+^CD45RA^-^, effector memory: CD27^-^CD45RA^-^); and (D), monocytes (hCD3^-^hCD19^-^hCD4^+^). FSC: forward scatter. SSC: side scatter.
(TIF)

S1 Data Excel file containing numerical data used to generate Figs 1B–1G, 2B–2G, 3–5, S1–S5, S6B and S6D. (XLSX)
