# Supplementary figures and images for: Transient CD4+ T cell depletion during suppressive ART reduces the HIV reservoir in humanized mice
Source: PLoS Pathog. 2023 Dec 6;19(12):e1011824. doi: 10.1371/journal.ppat.1011824 (PMC10699604; doi:10.1371/journal.ppat.1011824)

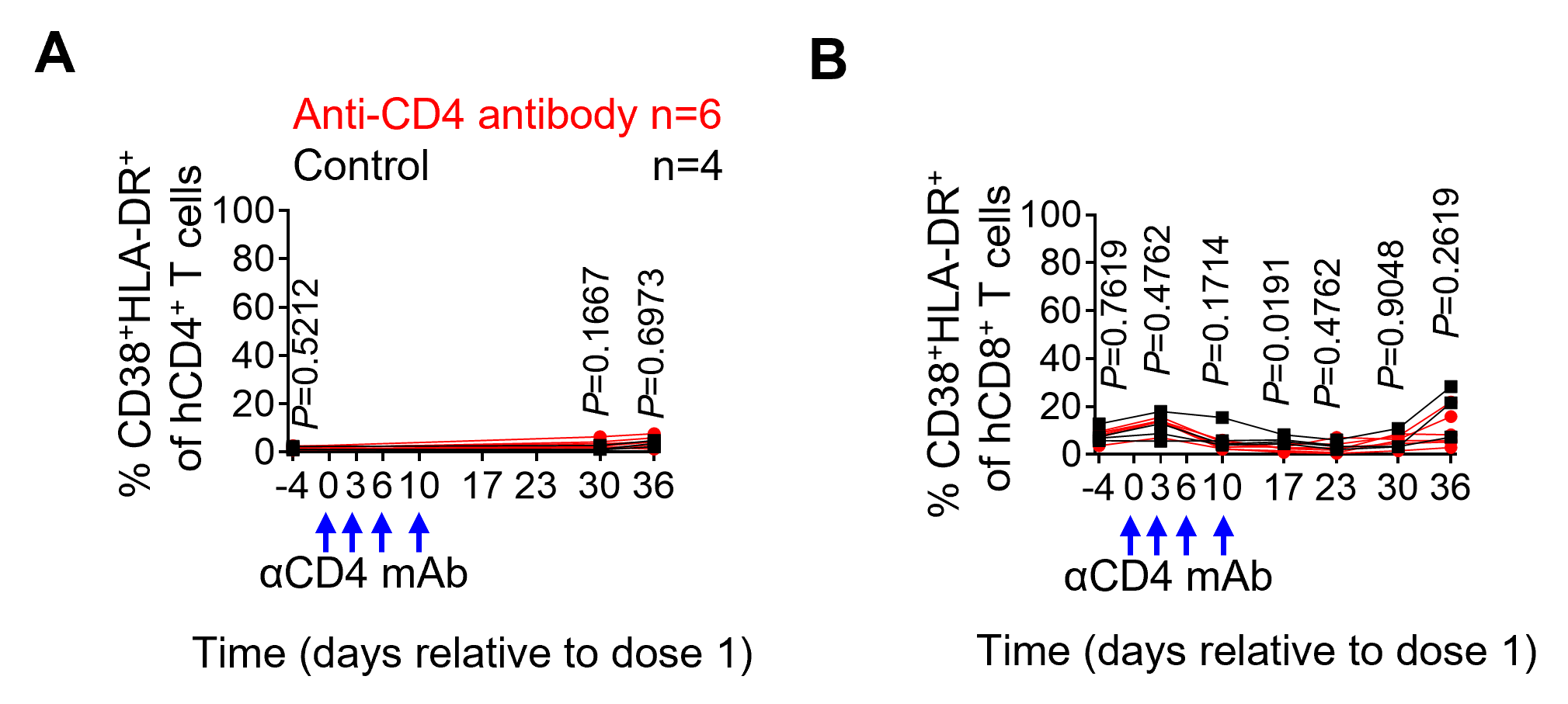

Supplement: S1 Fig — The frequency of activated (CD38+HLA-DR+) CD4+ (A) and CD8+ (B) T cells in the peripheral blood was longitudinally determined by flow cytometry. Blue arrows in A and B show the timing of 4 anti-CD4 antibody administrations to anti-CD4 antibody treated animals. Anti-CD4 antibody treated animals (n = 6) are shown in red; control animals (n = 4) are shown in black. Data are expressed as mean ± SEM. Statistical analyses were performed using unpaired two-sided Mann–Whitney U-tests. Statistical significance was considered when P < 0.05. (TIF) [file ppat.1011824.s001.tif]

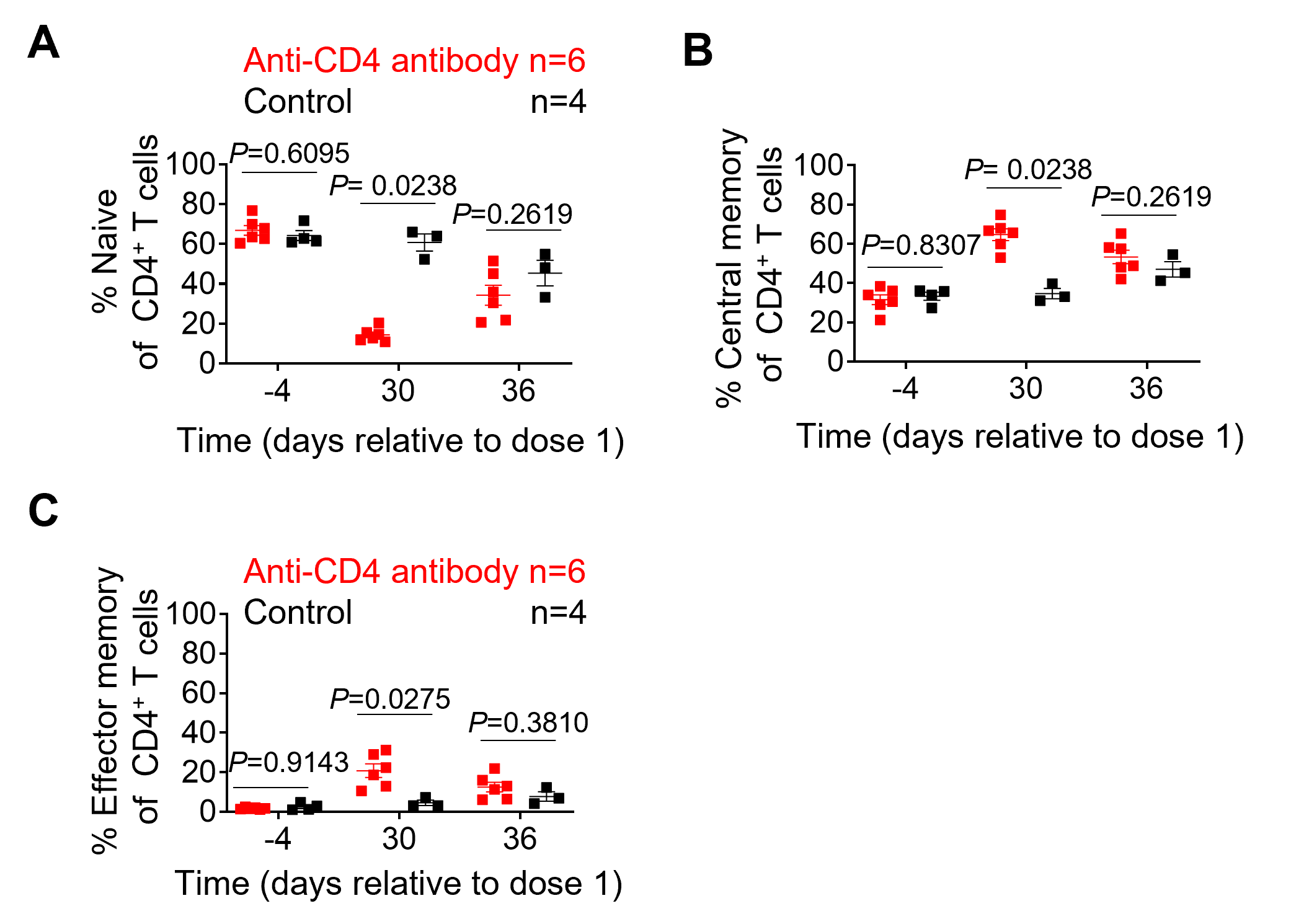

Supplement: S2 Fig — The frequency of naïve (A, CD27+CD45RA+), central memory (B, CD27+CD45RA-), and effector memory (C, CD27-CD45RA-) CD4+ T cells in the peripheral blood was longitudinally determined by flow cytometry. Blue arrows in A show the timing of 4 anti-CD4 antibody administrations to the anti-CD4 antibody treatment group. Anti-CD4 antibody treated animals (n = 6) are shown in red; control animals (n = 4) are shown in black. Data are expressed as mean ± SEM. Statistical analyses were performed using unpaired two-sided Mann–Whitney U-tests. Statistical significance was considered when P < 0.05. (TIF) [file ppat.1011824.s002.tif]

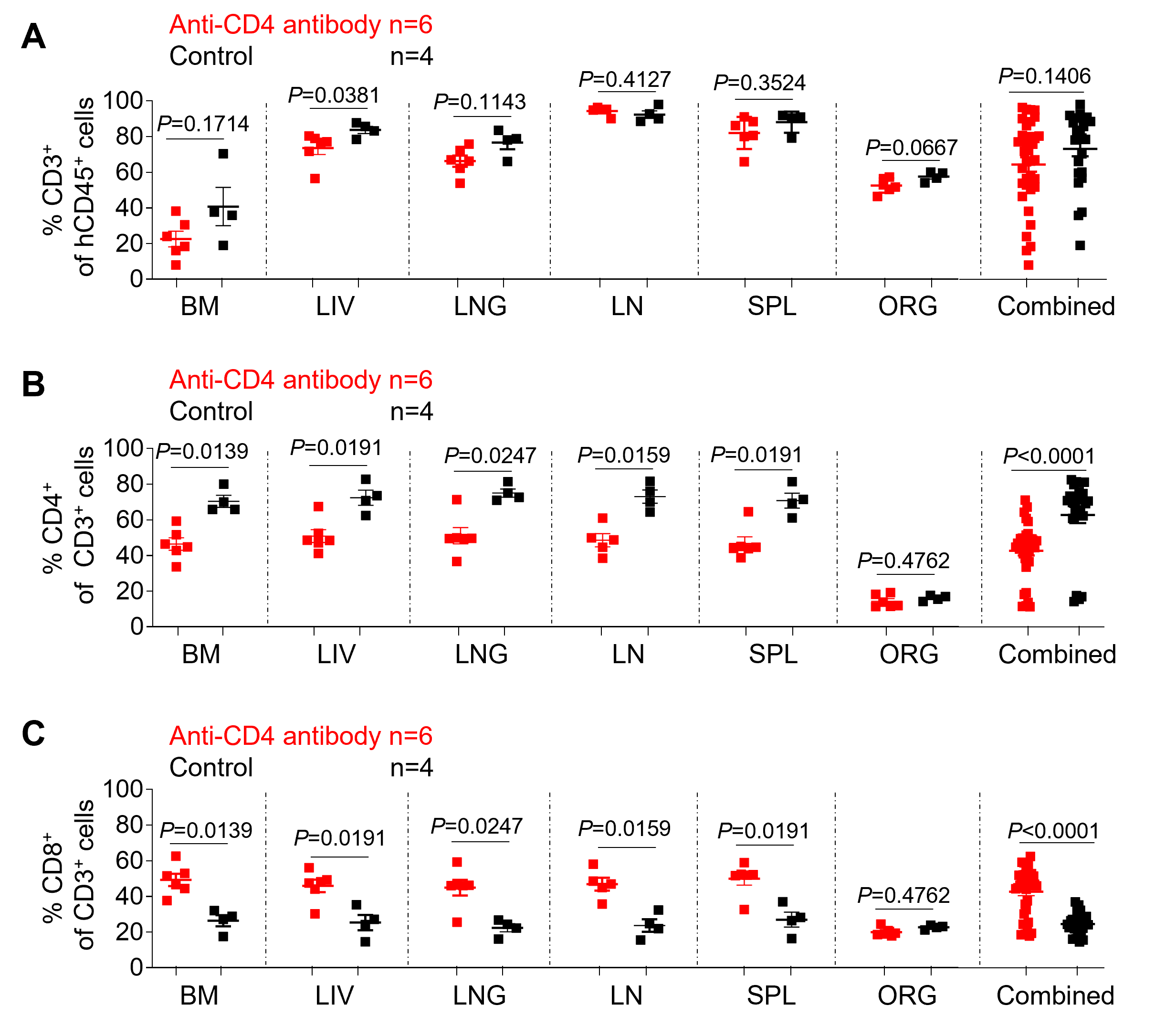

Supplement: S3 Fig — The frequency of human CD3+ T cells (A), CD4+ T cells (B) and CD8+ T cells (C) in tissues of anti-CD4 antibody-treated and control animals was determined by flow cytometry at necropsy. BM, bone marrow; LIV, liver; LNG, lung; LN, lymph nodes; SPL, spleen, ORG, human thymic organoid. Combined: all individual tissues from all animals are graphed together. Anti-CD4 antibody treated animals (n = 6) are shown in red; control animals (n = 4) are shown in black. Data are expressed as mean ± SEM. Statistical analyses were performed using unpaired two-sided Mann–Whitney U-tests. Statistical significance was considered when P < 0.05. (TIF) [file ppat.1011824.s003.tif]

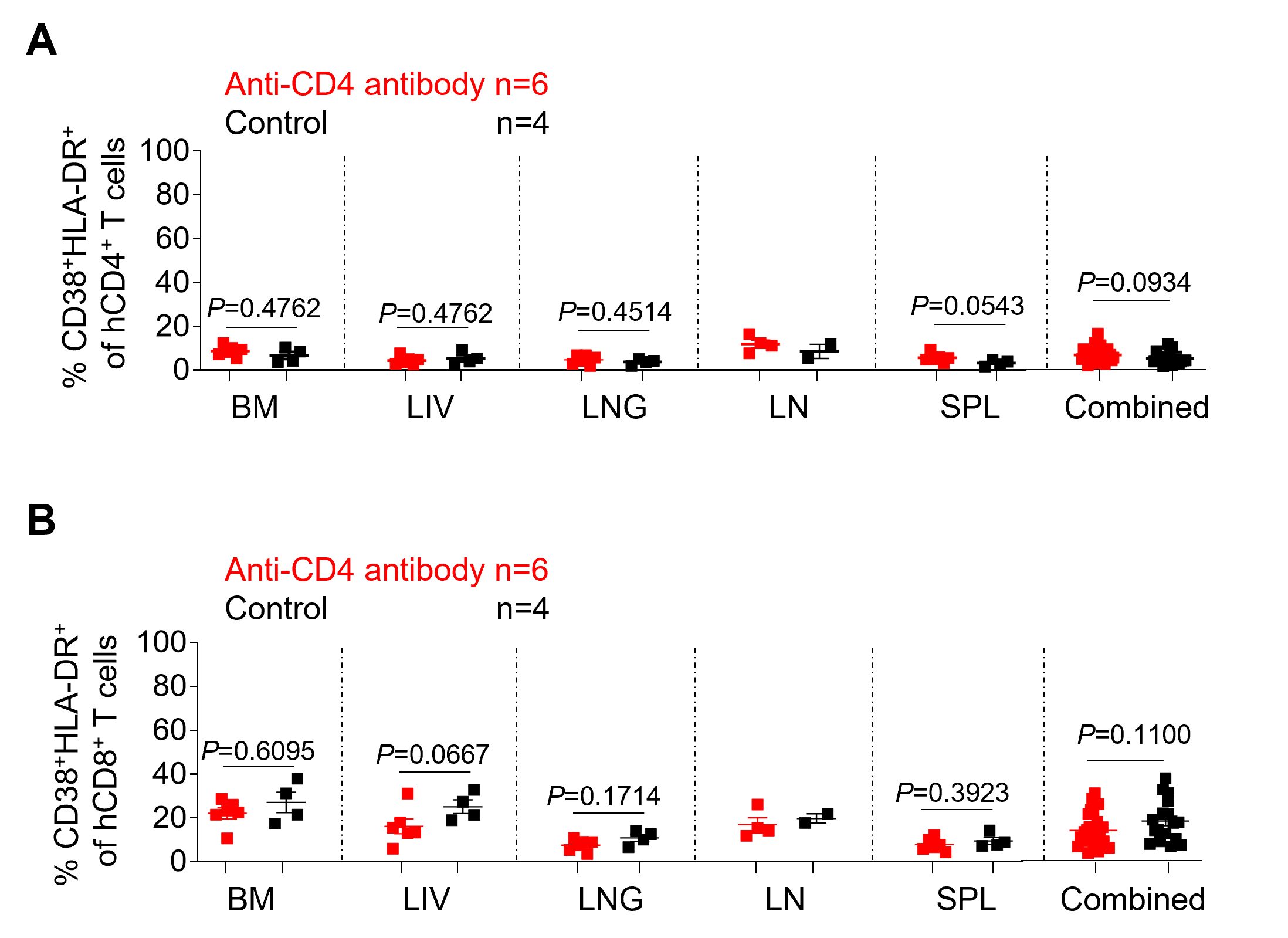

Supplement: S4 Fig — The frequency of activated (CD38+HLA-DR+) CD4+ (A) and CD8+ (B) T cells was determined by flow cytometry at necropsy. BM, bone marrow; LIV, liver; LNG, lung; LN, lymph nodes; SPL, spleen, ORG, human thymic organoid. Combined: all individual tissues from all animals are graphed together. Anti-CD4 antibody treated animals (n = 6) are shown in red; control animals (n = 4) are shown in black. Data are expressed as mean ± SEM. Statistical analyses were performed using unpaired two-sided Mann–Whitney U-tests. Statistical significance was considered when P < 0.05. (TIF) [file ppat.1011824.s004.tif]

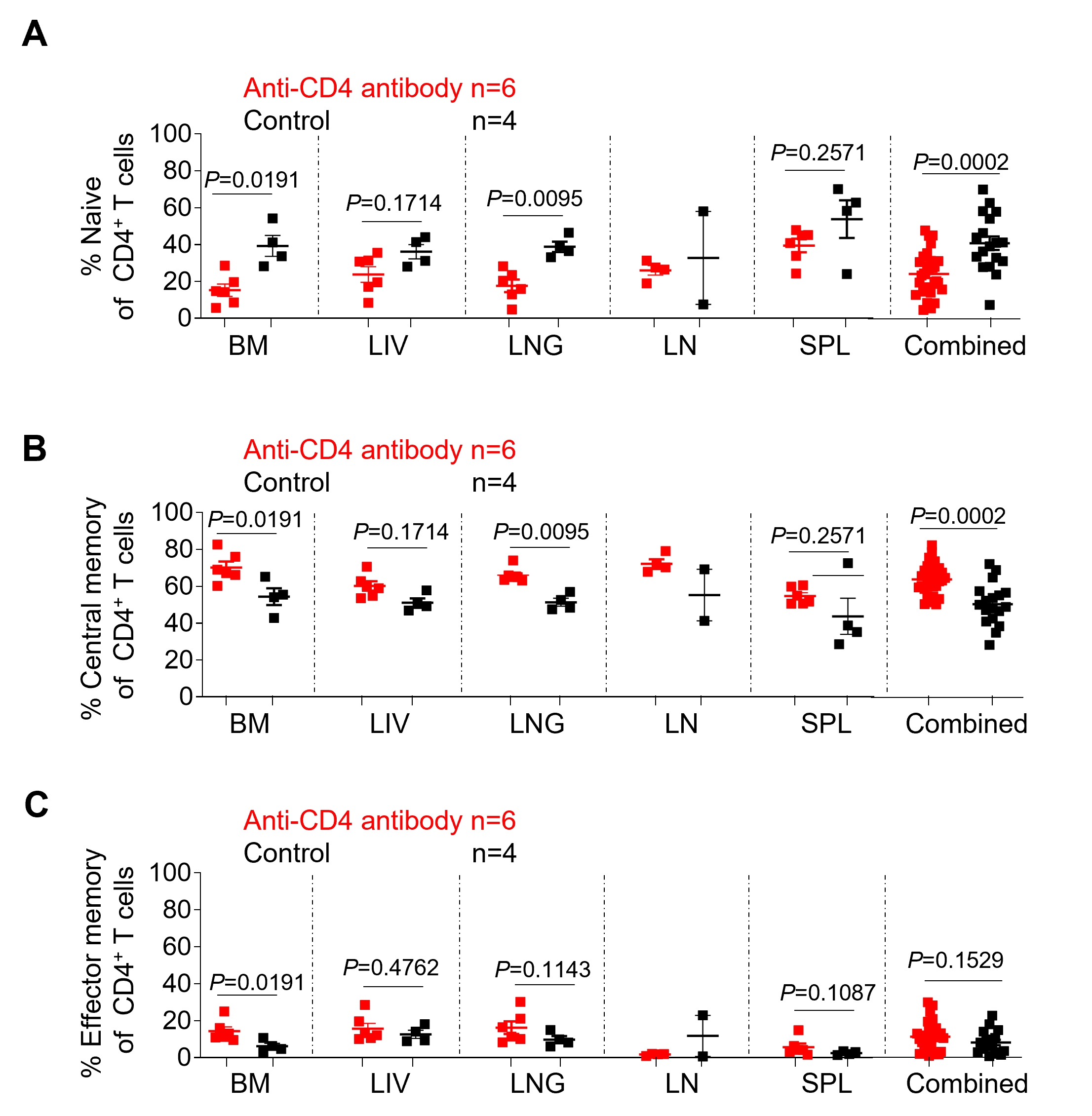

Supplement: S5 Fig — The frequency of naïve (A, CD27+CD45RA+), central memory (B, CD27+CD45RA-), and effector memory (C, CD27-CD45RA-) CD4+ T cells from anti-CD4 antibody-treated and control animals was determined by flow cytometry at necropsy. BM, bone marrow; LIV, liver; LNG, lung; LN, lymph nodes; SPL, spleen, ORG, human thymic organoid. Combined: all individual tissues from all animals are graphed together. Anti-CD4 antibody treated animals (n = 6) are shown in red; control animals (n = 4) are shown in black. Data are expressed as mean ± SEM. Statistical analyses were performed using unpaired two-sided Mann–Whitney U-tests. Statistical significance was considered when P < 0.05. (TIF) [file ppat.1011824.s005.tif]

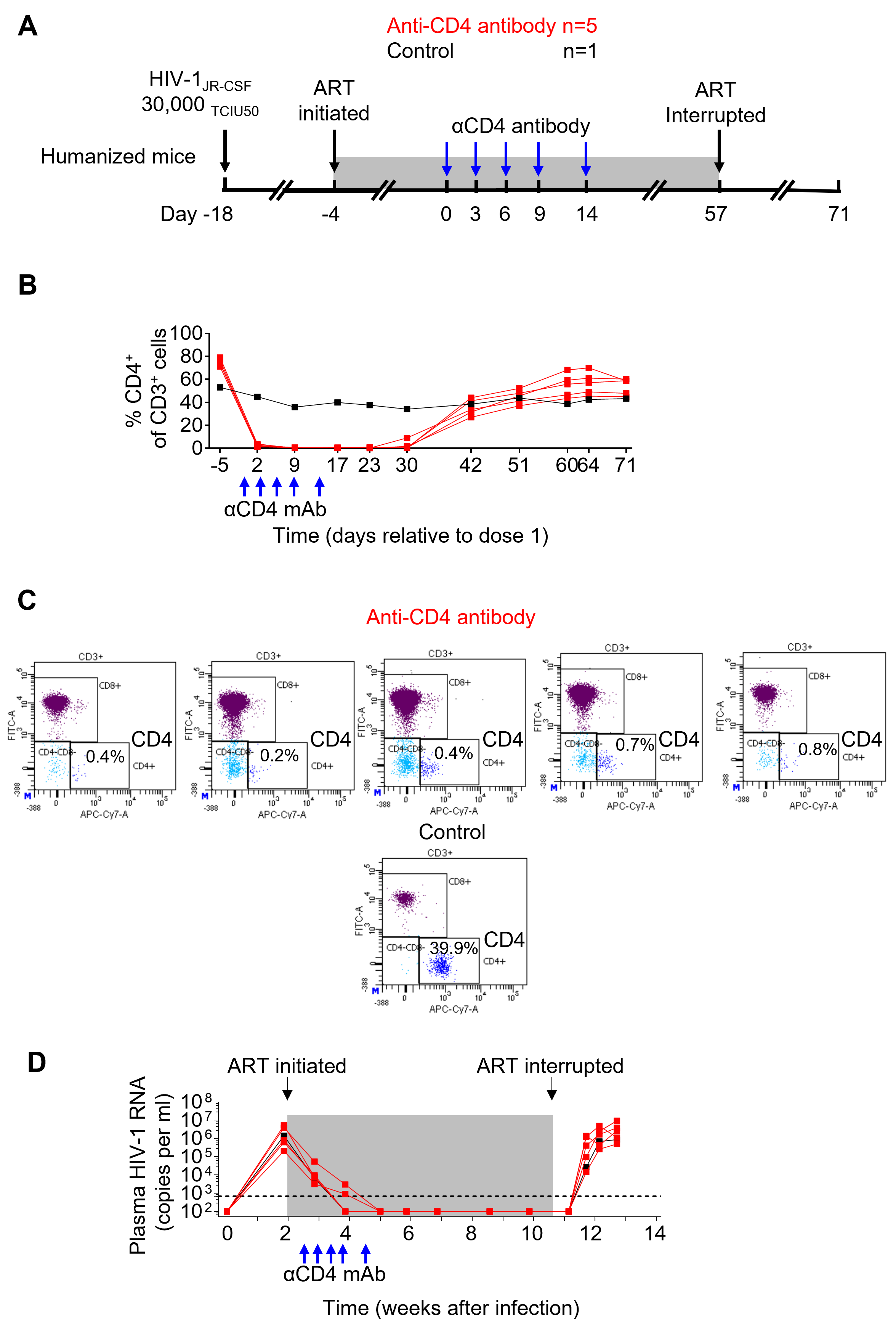

Supplement: S6 Fig — (A) Experimental design of HIV infection and anti-CD4 antibody treatment in humanized mice. (B) The frequency of CD4+ T cells in the peripheral blood was longitudinally monitored by flow cytometric analysis. (C) Flow plots showing the fraction of CD4+ T cells in anti-CD4 antibody-treated (top) and control (bottom) mice. (D) Plasma viral load (HIV-RNA copies/ml) was quantified in longitudinal plasma samples using a qRT-PCR assay following HIV-1 infection. Blue arrows show the timing of 5 anti-CD4 antibody administrations (6 mg/kg) to the anti-CD4 antibody treatment group. Anti-CD4 antibody treated animals (n = 5) are shown in red; control animals (n = 1) are shown in black. (TIF) [file ppat.1011824.s006.tif]

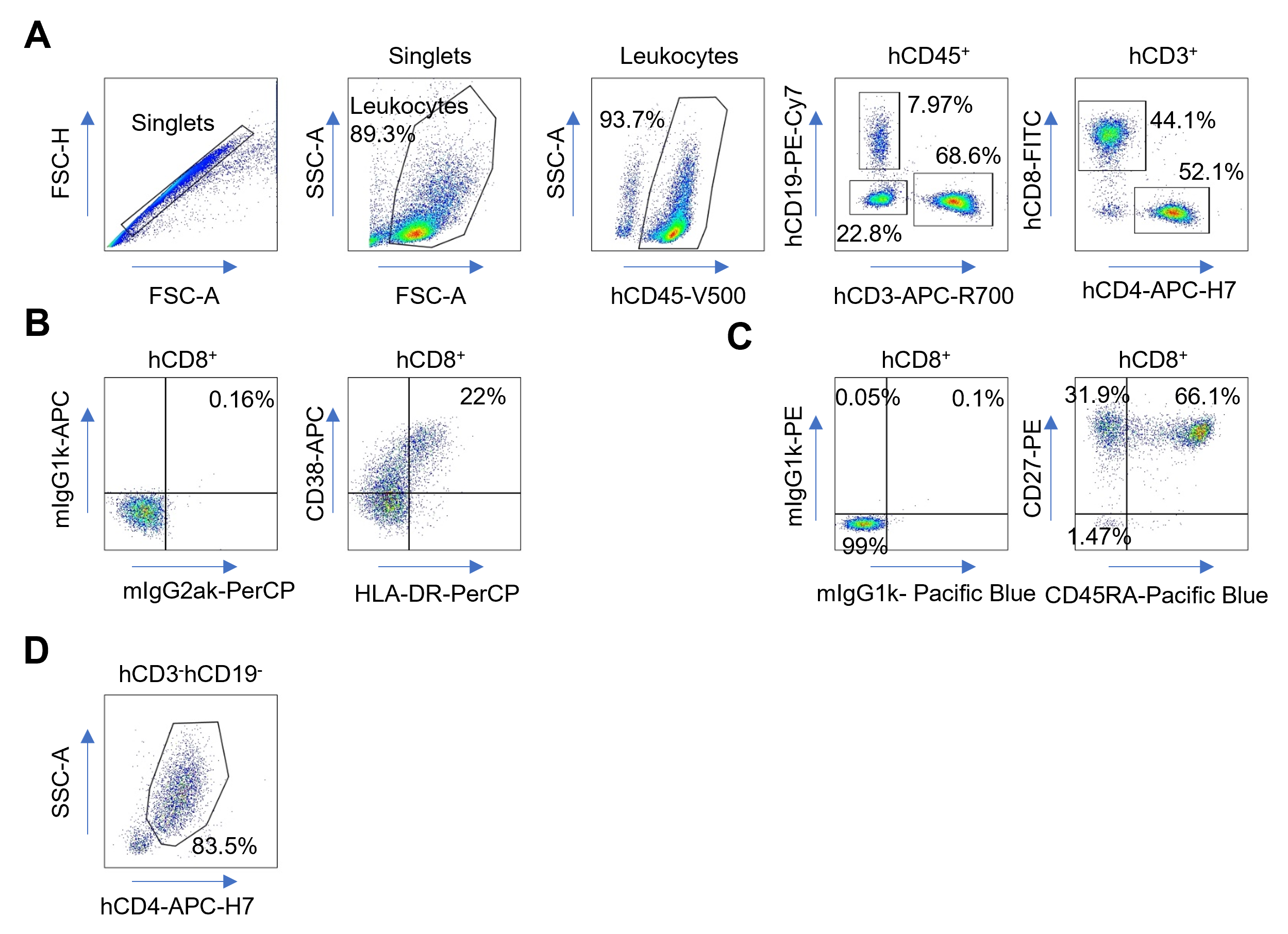

Supplement: S7 Fig — Shown are gating strategies for the analysis of (A), human immune cell subsets (hCD45+CD3+: T cells, hCD45+CD19+: B cells); (B), T cell activation (CD38+HLA-DR+); (C), T cell subsets (naïve: CD27+CD45RA+, central memory: CD27+CD45RA-, effector memory: CD27-CD45RA-); and (D), monocytes (hCD3-hCD19-hCD4+). FSC: forward scatter. SSC: side scatter. (TIF) [file ppat.1011824.s007.tif]
